# Behavioral responses to noxious stimuli shape the perception of pain

**DOI:** 10.1038/srep44083

**Published:** 2017-03-09

**Authors:** Elisabeth S. May, Laura Tiemann, Paul Schmidt, Moritz M. Nickel, Nina Wiedemann, Christian Dresel, Christian Sorg, Markus Ploner

**Affiliations:** 1 Department of Neurology and TUM-Neuroimaging Center, Technische Universität München, 81675 Munich, Germany; 2 Department of Statistics, Ludwig-Maximilians-Universität München, 80539 Munich, Germany; 3 Center for Neuroscience, The Feinstein Institute for Medical Research, Manhasset, NY 11030, USA; 4 Departments of Neuroradiology and Psychiatry and TUM-Neuroimaging Center, Technische Universität München, 81675 Munich, Germany

## Abstract

Pain serves vital protective functions. To fulfill these functions, a noxious stimulus might induce a percept which, in turn, induces a behavioral response. Here, we investigated an alternative view in which behavioral responses do not exclusively depend on but themselves shape perception. We tested this hypothesis in an experiment in which healthy human subjects performed a reaction time task and provided perceptual ratings of noxious and tactile stimuli. A multi-level moderated mediation analysis revealed that behavioral responses are significantly involved in the translation of a stimulus into perception. This involvement was significantly stronger for noxious than for tactile stimuli. These findings show that the influence of behavioral responses on perception is particularly strong for pain which likely reflects the utmost relevance of behavioral responses to protect the body. These observations parallel recent concepts of emotions and entail implications for the understanding and treatment of pain.

Pain is commonly defined as *an unpleasant sensory and emotional experience associated with actual or potential tissue damage*^1^. Pain has, thus, been mostly conceptualized as a perceptual phenomenon. However, the crucial protective function of pain depends on appropriate behavioral responses rather than on perception. The precise relationship between noxious stimuli, pain perception and behavioral responses is, however, not fully clear yet. Common models of stimulus-perception-behavior relationships in the somatosensory domain^2^ suggest that such behavioral responses result from a percept which, in turn, results from a stimulus. In view of the utmost relevance of protective behavioral responses for pain, we considered a view in which behavioral responses are in part directly elicited by the stimulus and then themselves shape pain perception. Such a partial independence of behavioral responses from perceptual processes could serve the protective functions of pain particularly fast and efficiently. It would, moreover, be consistent with models which emphasize an important role of motivational^3^,^4^,^5^ and motor^6^ processes for pain and recent concepts of emotions^7^,^8^,^9^. However, direct experimental evidence for such a role of behavioral responses for the perception of pain is lacking so far. We therefore explored the relationship between stimulus, perception and behavioral responses in a simple experiment in which healthy human subjects performed a reaction time task and provided perceptual ratings of noxious and tactile stimuli. As hypothesized, our results of a multi-level moderated mediation analysis^10^ show that behavioral responses are significantly and pain-specifically involved in the translation of a noxious stimulus into the perception of pain.

## Results

**Figure 1:**
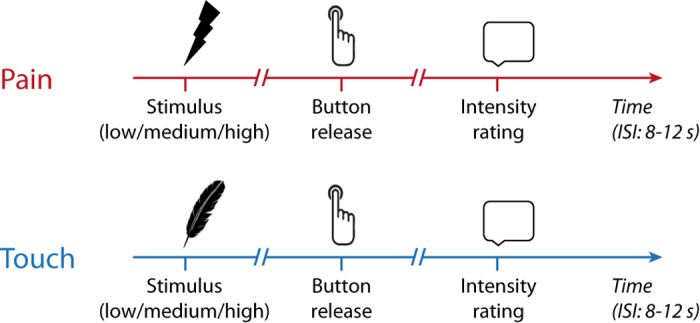
Paradigm. Pain and touch stimuli of varying intensity were applied to the right hand of healthy human subjects. Pain stimuli were brief cutaneous laser stimuli which selectively activate nociceptive afferents without activating tactile afferents. Touch stimuli were v.Frey-filaments applied by a computer-controlled device for standardized somatosensory stimulation. Reaction times were measured as button releases with the stimulated hand. Perceptual ratings were obtained on numerical rating scales from 0–100. ISI, inter-stimulus interval.

**Figure 2:**
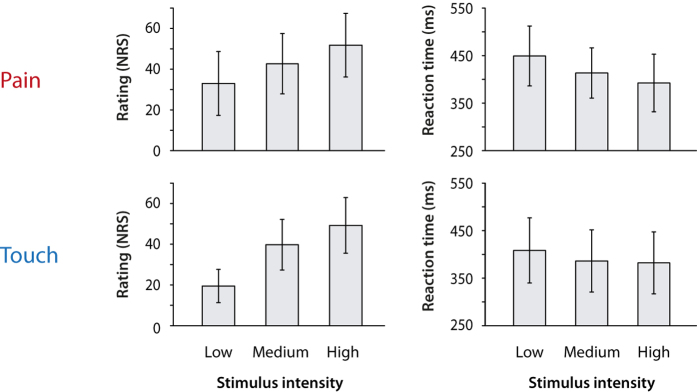
Perceptual ratings and reaction times to pain and touch stimuli. Mean ratings and reaction times for pain and touch stimuli of low, medium and high intensities are shown. Error bars indicate the standard deviation of individual means, i.e., they reflect the variability across subjects. Ratings increased and reaction times decreased with increasing intensity of pain and touch stimuli. ms, milliseconds; NRS, numerical rating scale.

To assess and compare the relationships between stimulus intensity, behavioral responses and perception for pain and touch, we performed a simple experiment. We applied brief painful heat and non-painful touch stimuli at three levels of individually determined stimulus intensities to the right hand of 55 healthy human subjects (Fig. 1). Subjects were instructed to react as fast as possible to each stimulus by releasing a button with the stimulated hand. Reaction times were taken as measures of behavioral responses to the stimuli. Subjects were further instructed to provide ratings of pain/touch intensity for each stimulus on a numerical rating scale (NRS) from 0 to 100 subsequent to the button release. Pain and touch ratings were taken as measures of perception. We then investigated the relationships between stimulus intensity, reaction times and perceptual ratings for both modalities (Fig. 2). As expected, linear mixed models showed that stronger stimuli yielded higher perceptual ratings (pain: *β* = 9.5, *p* < 0.001; touch: *β* = 14.9, *p* < 0.001) and shorter reaction times for pain (*β* = −25.1, *p* < 0.001) and touch stimuli (*β* = −12.8, *p* < 0.001). The *β*-values indicate that increasing the stimulus intensity by one level led on average to an increase of perceptual ratings of NRS 9.5 for pain and NRS 14.9 for touch stimuli and a decrease of reaction times of 25 ms for pain and 13 ms for touch stimuli.

**Figure 3:**
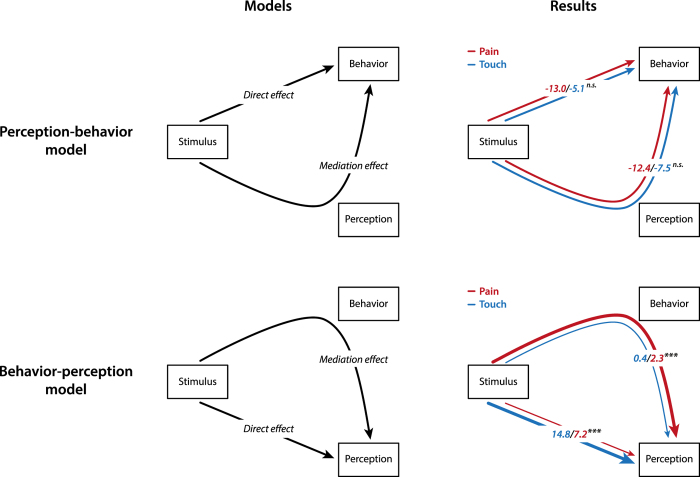
Moderated multi-level mediation analyses of the relationships between stimulus, behavior and perception. *Left*, The perception-behavior model reflecting the traditional view of perception-behavior relationships (upper panel) and the behavior-perception model reflecting an extension of the traditional view of relationships (lower panel). *Right*, Results of the moderated multi-level mediation analyses for both models for pain and touch. All effects of both models and modalities were by themselves significant with the exception of the direct effect in the perception-behavior model for touch stimuli. Significant differences between modalities (pain vs. touch) are marked by asterisks and differences in thickness of arrow lines. In the perception-behavior model (upper panel), the direct effect of stimulus on behavior and the mediation effect of perception did not differ between modalities. In the behavior-perception model (lower panel), the mediation effect of behavior was significantly stronger for pain than for touch. In contrast, the direct effect was significantly stronger for touch than for pain. All effects are quantified in original units (milliseconds for behavior in the upper panel, ratings on the numerical rating scales for perception in the lower panel) so that coefficients reflect the estimated average effects of a one level stimulus intensity increase on the respective dependent variable. n.s., not significant; ***p < 0.001.

To further explore the relationships between stimulus intensity, behavioral responses and perception, we performed multi-level moderated mediation analyses^10^. Mediation analysis is a statistical approach which quantifies the involvement of an intervening variable called mediator in the effects of an independent variable on a dependent variable. To this end, the total effect of the independent variable is divided into a direct effect which measures the immediate effect on the dependent variable, and a mediation effect which specifies the part that is passed indirectly via the mediator. We specifically tested mediation effects for two different models (Fig. 3, left). We assessed a *perception*-*behavior model* based on the view in which perception mediates the effects of stimulus intensity on behavioral responses (Fig. 3, left, upper panel). In addition, we tested a second model reflecting an extension of this view. In this *behavior*-*perception model*, behavioral responses mediate the effects of stimulus intensity on perception (Fig. 3, left, lower panel). As we considered behavioral responses and perception as interacting processes, we expected significant relationships between stimulus intensity, behavior and perception in both models. However, considering the utmost relevance of protective behavioral responses for pain, we hypothesized that the mediation effect of behavior in the stimulus-perception relationship would be significantly stronger for painful than for non-painful stimuli. In the logic of mediation analyses, that would mean that modality (pain vs. touch) moderates the mediation effect in the behavior-perception model. We, thus, did not perform formal model comparisons of the two models but assessed moderation effects within both models.

In the perception-behavior model (Fig. 3, right, upper panel), the mediation effects (ME) of perception in the stimulus-behavior relationship (pain: *β*_*ME*_ = −12.4, 95% confidence interval (CI): [−15.3, −9.6]; touch: *β*_*ME*_ = −7.5, CI: [−11.4, −4.1]) and the direct effects (DE) of stimulus intensity on behavior (pain: *β*_*DE*_ = −13.0, CI: [−17.3, −8.7]; touch: *β*_*DE*_ = −5.1, CI: [−9.7, 0.1]) did not differ across modalities. Thus, perception was similarly involved in the effects of stimulus intensity on behavioral responses for touch and pain. In contrast, in the behavior-perception model, the mediation effects of behavior differed significantly between touch and pain (Fig. 3, right, lower panel). Specifically, behavior mediated the stimulus-perception relationship significantly stronger for pain (*β*_*ME*_ = 2.3, CI: [1.7, 3.0]) than for touch (*β*_*ME*_ = 0.4, CI: [0.2, 0.6]). In contrast, the direct effect of stimulus intensity on perception was stronger for touch (*β*_*DE*_ = 14.8, CI: [13.3, 16.4]) than for pain (*β*_*DE*_ = 7.2, CI: [5.9, 8.6]). In other words, the total effect of stimulus intensity on pain perception breaks down into a NRS 7.2 direct effect of stimulus intensity on perception and a NRS 2.3 mediation effect of behavior. For touch stimuli, the total effect breaks down into a NRS 14.8 direct effect and a NRS 0.4 mediation effect. Thus, the proportion of the mediation effect of behavior to the total effect of stimulus intensity on perception was significantly higher for pain (24.3%, CI: [18.4, 32.2]) than for touch (2.3%, CI: [1.1, 3.6]), indicating that behavioral responses are significantly and pain-specifically involved in the translation of a noxious stimulus into the perception of pain.

**Figure 4:**
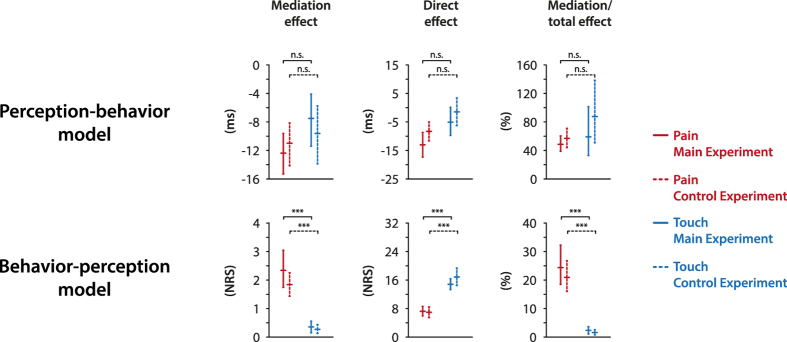
Replication of results in a control experiment. Mediation effects, direct effects and the proportion of mediation effects to total effects and their 95% confidence intervals are shown for pain and touch in the perception-behavior (upper row) and the behavior-perception (lower row) model. Results from the main experiment (with block-wise presentation of pain and touch stimuli) are depicted using solid lines, results from the control experiment (with a mixed presentation of pain and touch stimuli within each block) using dashed lines. As in Fig. 3, all effects are depicted in original units and significant differences between modalities are marked. The control experiment fully replicated the pattern of results from the main experiment. ms, milliseconds; NRS, numerical rating scale; n.s., not significant; ***p < 0.001.

To replicate our results and test whether these findings can be explained by a presentation of pain and touch stimuli in different blocks and by modality-specific expectations, we performed a control experiment with a randomized order of pain and touch stimuli in 35 healthy human subjects. The results fully replicated the results of the main experiment (Fig. 4). In the perception-behavior model, mediation effects (pain: *β*_*ME*_ = −11.0, CI: [−14.2, −8.1]; touch: *β*_*ME*_ = −9.6, CI: [−13.9, −5.7]) and direct effects (pain: *β*_*DE*_ = −8.3, CI: [−11.7, −5.0]; touch: *β*_*DE*_ = −1.5, CI: [−6.3, 3.4]) did not differ across modalities. In the behavior-perception model, behavioral responses again mediated the stimulus-perception relationship significantly stronger for pain than for touch (pain: *β*_*ME*_ = 1.8, CI: [1.4, 2.3]; touch: *β*_*ME*_ = 0.3, CI: [0.1, 0.4]). The findings further confirmed a significantly stronger direct effect of stimulus intensity on perception for touch than for pain (pain: *β*_*DE*_ = 7.0, CI: [5.4, 8.5]; touch: *β*_*DE*_ = 16.8, CI: [14.5, 19.3]). Finally, the proportion of the mediation effect of behavior to the total effect of stimulus intensity on perception was again significantly higher for pain (21.0%, CI: [16.0, 26.8]) than for touch (1.5%, CI: [0.7, 2.6]).

## Discussion

In the present study, we investigated the relationships between stimulus intensity, behavioral responses and perception and compared these relationships for pain and touch. Multi-level moderated mediation analyses revealed that behavioral responses are involved in the translation of a stimulus into perception. This effect was substantially stronger for painful than for non-painful stimuli indicating a pain-specific contribution of behavioral responses to perception. These findings advocate a conception of pain in which behavioral responses to noxious events are not fully dependent on perceptual processes but themselves shape pain perception.

Common models of stimulus-perception-behavior relationships in the somatosensory domain^2^ suggest that a stimulus induces a percept which, in turn, induces a behavioral response. However, neurophysiological recordings in the tactile domain already questioned a strict separation of perceptual and motor processes^11^. The present findings reveal that a strictly serial perception-behavior sequence is even less appropriate for pain. Instead, our findings show that perception and behavior are dynamically interacting processes. As indicated by significant mediation effects in both models for both modalities, such mutual interactions likely exist in all sensory modalities. Although mediation analysis does not necessarily imply causal relationships^12^, our findings indicate that the balance of these behavior-perception interactions differs significantly between pain and touch with a stronger role of behavior for the perception of pain.

The fact that noxious stimuli can induce behavioral responses in part independently from perceptual processes might serve the protective function of pain particularly well. Pain has an *inherent* threat value, whereas non-painful events signal threat only under specific conditions, e.g. when associations between sensory events and threat have been learned. This inherent threat value of pain demands a particularly fast and efficient translation of noxious stimuli into behavioral responses to avoid or limit injury and promote recovery^5^ which is supported by the current results. Importantly, in the present experiment, the behavioral responses did not have a true protective function; they did not prevent future stimuli or reduce their intensity. The observed difference in the role of behavior for perception does, thus, not reflect the momentary adaptive function of the behavioral response but a hardwired, intrinsic difference in the organization of the stimulus-perception-behavior relationships between pain and other modalities. Behavioral responses with true adaptive functions might exert even stronger effects on perception than the moderate effects observed under the present experimental conditions.

Anatomically, a partial independence of behavioral responses from perception might be implemented by a largely parallel organization of pain pathways. Ascending pain pathways project in parallel via the thalamus and the brainstem to somatosensory^13^,^14^, motor^15^, insular^16^ and cingulate^17^ cortices as well as to subcortical areas including the amygdala^18^,^19^ and the nucleus accumbens^19^,^20^. In particular, projections to the amygdala and the nucleus accumbens which have been related to the motivation and initiation of defensive behavior^7^,^21^ might be instrumental for reactions and actions in response to noxious stimuli. Such direct thalamic and brainstem projections to subcortical areas also exist for non-painful stimuli from different modalities^22^,^23^. However, the functional relevance of these direct pathways which bypass extensive sensory processing likely depends on the threat value of the stimuli^22^. Due to the *inherent* threat value of pain, these direct pathways might be particularly relevant for the processing of noxious stimuli and ensuing protective responses^24^,^25^.

The precise mechanisms of the difference in the stimulus-perception-behavior relationship between pain and touch remain to be clarified. Pain is inherently associated with a high salience and negative valence^26^ which is confirmed by higher salience and unpleasantness ratings for pain than touch stimuli in the current study. From the present findings, we can therefore not disentangle the differential contributions of modality, salience and valence to the observed effects. Moreover, the translation of a noxious stimulus into a behavioral response comprises the motivation, preparation and execution of behavioral responses. Whether and how these processes are differentially involved in the effects of behavior on perception remains to be demonstrated. In the light of previous conceptions of pain^3^,^4^,^5^, we hypothesize that motivational processes play an important role in shaping pain perception. Such a relevance of motivational aspects of pain has increasingly attracted attention^20^,^27^,^28^,^29^ and the present findings are well compatible with these conceptions.

Our view of pain in which behavioral responses shape pain perception parallels recent concepts of emotions^7^,^8^,^9^. It has been proposed that emotions essentially reflect functions and brain circuits serving survival. These circuits coordinate and monopolize brain resources for reactions and actions aimed at promoting and protecting the individual. In this concept, conscious feelings are no longer understood as the exclusive driving force of behavioral responses but are partially shaped by activation of survival circuits and/or the resulting behavioral responses. These views represent extensions of earlier emotion theories^30^ which emphasize the role of body feedback for emotional feelings. If we conceptualize pain perception as an emotional feeling, our findings are well compatible with these views. The motivation and/or execution of behavioral responses to threatening, e.g. painful, stimuli might be implemented by activity of survival circuits which is not only the result but also the substrate of an emotional feeling, i.e. of the perception of pain.

Clinical observations lend further support to the crucial relevance of the behavior-perception relationship for pain. Psychological factors like passive coping, low self-efficacy and helplessness^31^ as well as abnormal behavioral patterns^6^ are important aspects of the pathology of chronic pain which can be considered as changes in the motivation and/or execution of behavioral responses to pain. The paradigm of the present study might represent a simple behavioral tool to gain insight into these processes. Our approach allows for a quantitative assessment of the mutual effects of pain behavior and pain perception which might be pathologically altered as a cause, correlate or consequence of chronic pain. Moreover, our findings might also be relevant for the treatment of pain. In a traditional, perception-centered concept of pain, pain therapy focuses on the alleviation of pain perception. The present, extended view which considers pain behavior as an important determinant of pain perception implies that the treatment of motivational deficits and pain behavior might ultimately also relieve pain perception. Such therapeutic approaches might differ from approaches directly targeting perception and might not only include behavioral interventions but also pharmacological approaches. In this regard, the dopaminergic system with its close association to motivation and action^21^ and emerging evidence for its involvement in chronic pain^32^,^33^ might play an interesting role.

In summary, the present approach provides quantitative insights into stimulus-perception-behavior relationships and their differences between pain and touch. The results reveal that behavioral responses to noxious stimuli shape the perception of pain which indicates that common models of stimulus-perception-behavior relationships in the somatosensory domain do not fully apply to pain. Instead, our findings advocate a concept of pain in which behavioral and perceptual processes are partially independent processes that shape each other. Such a view parallels current concepts of emotions and entails implications for the understanding and treatment of chronic pain.

## Materials and Methods

### Subjects

55 healthy human subjects (age 27 ± 6 yrs (mean ± standard deviation), 25 females) participated in the main experiment. 35 subjects (age 27 ± 5 yrs (mean ± standard deviation), 17 females) participated in a subsequent control experiment, 23 of which had already taken part in the main experiment. All subjects were right-handed and gave written informed consent. The study was approved by the ethics committee of the Medical Faculty of the Technische Universität München and conducted in conformity with the declaration of Helsinki.

### Paradigm

In the main experiment, pain and touch stimuli were applied to the dorsum of the subject’s right hand (Fig. 1). The subject’s task was to first react to each stimulus as fast as possible by releasing a button with the right index finger and then verbally rate each stimulus on a numerical rating scale (NRS) ranging from 0 to 100 and anchored at *no pain* and *maximum tolerable pain* for pain stimuli and at *no touch* and *maximum non*-*painful touch* for touch stimuli. An equal importance of a fast reaction and a precise rating was emphasized during instructions. Pain and touch stimuli were applied in 4 blocks with short breaks in between. In each block, only stimuli of one modality were delivered. The order of blocks (pain/touch/pain/touch or touch/pain/touch/pain) was counterbalanced across subjects. Within each block, stimulus intensity was pseudorandomly varied between three levels (low, medium, high; see below) with the restriction that not more than two stimuli of the same intensity were applied in a row. In each block, 17 pain or touch stimuli of each low, medium and high intensity (see below) were applied with a randomly varied inter-stimulus interval between 8 and 12 s, resulting in 51 trials per block and in a total of 102 pain and 102 touch trials for the whole experiment. Before the first and second blocks of each modality, 15 and 6 practice trials were performed, respectively, which were not included in the final analysis. The experiment was performed with eyes closed.

In the control experiment, pain and touch stimuli were not applied in modality-specific blocks but applied pseudorandomly within blocks. Thus, each block contained both pain and touch stimuli, controlling for modality-specific expectations. The order of stimuli in each block was again randomized, now with the additional restriction that not more than three stimuli of the same modality were administered in a row. Again, four blocks of stimulation were performed, now each consisting of 9 pain and touch stimuli at low, medium and high intensity, resulting in 54 trials per block and a total of 108 pain and 108 touch trials for the whole experiment. 18 practice trials were performed before the first, 6 practice trial before each subsequent block. After reacting to and before rating each stimulus, subjects were asked to verbally identify the stimulus modality, i.e. pain or touch. All other parameters matched the main experiment.

Stimulus presentation and timing was controlled using Matlab (Mathworks, Natick, MA, USA) and the Psychophysics Toolbox (http://psychtoolbox.org/). Responses by the subject were given using a custom-built response box (MES Forschungssysteme GmbH, Gilching, Germany), allowing an acquisition of response timing with millisecond accuracy.

After the last blocks of both the main and the control experiments, ratings of the average stimulus intensity, unpleasantness and salience across all trials were obtained for pain and touch stimuli using visual analogue scales ranging from not intense/unpleasant/salient to highly intense/unpleasant/salient. In line with the inherent threat value of pain, the results confirmed that pain stimuli were rated as more intense, more salient and more unpleasant than touch stimuli (Wilcoxon signed rank tests; all p-values < 0.001 in both experiments). In addition, visual analogue scale ratings of the difficulty of the combined task of reacting to and rating the stimuli (very easy to very difficult) and task preference (very focused on the reaction to very focused on the rating) were obtained for touch and pain. Ratings for task difficulty and task preference did not differ significantly between pain and touch (Wilcoxon signed rank tests; p > 0.05 in both experiments).

### Stimuli

Pain stimuli were brief laser heat stimuli which selectively activate nociceptive afferents without concomitant activation of tactile fibers^34^. Stimuli were administered using a Tm:YAG laser (Starmedtec GmbH, Starnberg, Germany) with a wavelength of 1960 nm, a pulse duration of 1 ms and a spot diameter of 5 mm. A distance pin mounted to the hand piece of the laser device ensured a constant distance between skin surface and laser device. Stimulation site was slightly changed after each stimulus to avoid tissue damage.

Touch stimuli were applied using an in house-developed device^35^ employing von Frey-filaments to deliver punctate tactile stimuli to a small area of the skin (≤1 mm^2^) with a high precision of the applied intensity and timing. Constant, logarithmically-scaled forces between 8 and 512 mN were used, stimulus duration was 80 ms.

For both the main and the control experiment, individual stimulation intensities were determined beforehand separately for pain and touch stimuli. The order (pain/touch or touch/pain) was counterbalanced across subjects. A regression analysis was used which related objective stimulation intensities to subjective ratings on the basis of 20 stimuli of random intensities of each modality. Low, medium and high stimulus intensities were selected, aiming at ratings of 30, 50 and 70 on the same NRS used during the experiments which ranged from 0 to 100 and anchored at *no pain* and *maximum tolerable pain* for pain stimuli and at *no touch* and *maximum non*-*painful touch* for touch stimuli. Maximally used stimulation intensities were 600 mJ and 512 mN for pain and touch stimuli, respectively. For the main experiment, mean (±standard deviation) stimulation intensities across subjects were 425 mJ (±81), 483 mJ (±75) and 541 mJ (±74) for pain stimuli and 123 mN (±69), 265 mN (±96) and 412 mN (±110) for touch stimuli of low, medium and high intensity. For the control experiment, mean (±standard deviation) stimulation intensities were 467 mJ (±49), 519 mJ (±46) and 571 mJ (±44) for pain stimuli and 133 mN (±68), 261 mN (±77) and 404 mN (±94) for touch stimuli of low, medium and high intensity.

### Analysis

Only trials with a rating between 1 and 100 on the respective NRS were used, ensuring that all analyzed noxious stimuli had elicited painful sensations and all tactile stimuli non-painful touch sensations. Furthermore, only trials with a reaction time between 150 ms and 650 ms were included. Painful laser stimuli activate both A-delta- and C-fibers, resulting in a bimodal reaction time distribution reflecting different conduction velocities and first and second pain perception (e.g., refs 36 and 37). To exclude trials with C-fiber activation only, a limit of 650 ms has been shown to be a useful cut-off for the upper limb (e.g., refs 37 and 38). For comparability, touch trials were selected accordingly. In the control experiment, only those trials in which the stimulus modality (pain/touch) was correctly identified were included.

For the main experiment, mean (±standard deviation) resulting trial numbers across subjects were 21 (±9), 29 (±6) and 32 (±4) for pain stimuli and 31 (±5), 32 (±4) and 32 (±4) for touch stimuli of low, medium and high intensity, respectively. For the control experiment, mean (±standard deviation) resulting trial numbers were 28 (±8), 33 (±4) and 34 (±3) for pain stimuli and 35 (±2), 35 (±2) and 35 (±2) for touch stimuli of low, medium and high intensity, respectively. Trial numbers were taken into account in all further analyses.

Relationships between stimulus intensity, reaction times and ratings were investigated using R^39^ and the *lme4*^40^ and *mediation*^41^ packages. Data from the main and the control experiment were analyzed separately but fully in parallel. For all analyses, stimulus intensity was treated as a numeric variable and centered around 0. Reaction times and ratings were z-transformed across all trials and subjects for pain and touch stimuli separately.

To explore the effects of stimulus intensity on ratings and reaction times, we fitted linear mixed models to the two combinations of variables (*stimulus intensities – reaction times and stimulus intensities – ratings*) using the *lmer* function, including the effect of stimulus modality (pain vs. touch) as additional regressor. Variations of effects at both the individual subject level (random effects) and across subjects (fixed effects) were modeled. These analyses yielded statistical estimates of the strengths of the relationships between each two variables for both pain and touch stimuli. For an easier grasp of the size of effects, beta weights resulting from the regression analyses were re-transformed back into originals units (NRS for ratings and ms for reaction times) by inverting the z-standardization of ratings and reaction times after the analysis. This resulted in a quantification of the effect of one level of stimulus intensity increase on ratings and reaction times expressed in original units.

Next, we aimed at going beyond the analysis of simple associations between stimulus intensities, reaction times and perceptual ratings for pain and touch. To this end, we applied moderated mediation analyses to our data^10^,^41^. Mediation analysis is a statistical approach which investigates the relationship between an independent variable *X*, a dependent variable *Y* and an intervening variable *M* called mediator. A variable *M* is a mediator, if *X* affects *Y* because *X* affects *M* and *M* affects *Y*. The total effect of *X* on *Y* in mediation analysis is composed of two effects (total effect = direct effect + mediation effect). The *direct effect* represents the effects of *X* on *Y* independent of the mediator. The *mediation effect* represents the effect of *X* on *Y* transmitted via the mediator, as described above. Moderated mediation analysis additionally quantifies how this mediation effect changes in the light of an additional covariate, the *moderator*.

In our case, we were interested in testing two different moderated mediation models (Fig. 3, left), both composed of the predicting variable *stimulus* (operationalized by three levels of stimulus intensity) and the two response variables *behavior* (measured by reaction times) and *perception* (measured by ratings). Importantly, the dependency of all effects on the moderating variable *modality* (pain or touch) was tested. The *perception*-*behavior model* (Fig. 3, left, upper panel) investigated the extent to which the effect of the stimulus (*X*) on behavior (*Y*) was mediated by perception (*M*). The *behavior*-*perception model* (Fig. 3, left, lower panel), investigated the extent to which the effect of the stimulus (*X*) on perception (*Y*) was mediated by behavior (*M*). Importantly, for both models, the magnitudes for direct and mediation effects as well as the proportion of the mediation effect relative to the total effect (mediation effect/total effect) were estimated and compared between the levels of the moderator, i.e. between pain and touch stimuli. In more detail, a set of linear mixed models was first fitted using the *lmer* function quantifying the conditional distribution of the respective mediating variable M given the manipulation of the stimulus X and the conditional distribution of the outcome variable *Y* given the mediating variable *M* and the stimulus *X*. As before, random and fixed effects were modeled. Based on these models, estimates of direct and mediation effects on both levels of the moderator modality were then computed using the *mediate* function. Statistical inferences were based on the quasi-Bayesian Monte Carlo method, 1000 simulations and the 95% confidence intervals of all estimates. Significance of the different effects (mediation effects, direct effects and proportion mediation to total effects) in each modality was inferred if the respective confidence interval did not include zero. Significance of modality differences was inferred from non-overlapping confidence intervals of the effects for pain and touch stimuli. For significantly different effects, exact p-values were then obtained by subtracting the Monte Carlo samples for both modalities and examining the resulting distribution of differences^41^. Again, for an easier grasp of the size of effects, all obtained coefficients were finally re-transformed back into originals units to quantify the average effect of a one level stimulus intensity increase across all trials and subjects. Raw data and the full R code can be obtained from the corresponding authors on request.

## Additional Information

**How to cite this article**: May, E. S. *et al*. Behavioral responses to noxious stimuli shape the perception of pain. *Sci. Rep.* **7**, 44083; doi: 10.1038/srep44083 (2017).

**Publisher's note:** Springer Nature remains neutral with regard to jurisdictional claims in published maps and institutional affiliations.
